# Gradiometer Using Separated Diamond Quantum Magnetometers

**DOI:** 10.3390/s21030977

**Published:** 2021-02-02

**Authors:** Yuta Masuyama, Katsumi Suzuki, Akira Hekizono, Mitsuyasu Iwanami, Mutsuko Hatano, Takayuki Iwasaki, Takeshi Ohshima

**Affiliations:** 1 Takasaki Advanced Radiation Research Institute, National Institutes for Quantum and Radiological Science and Technology, Takasaki, Gunma 370-1292, Japan; hatano.m.ab@m.titech.ac.jp (M.H.); ohshima.takeshi@qst.go.jp (T.O.); 2 Department of Electrical and Electronic Engineering, Tokyo Institute of Technology, Meguro, Tokyo 152-8552, Japan; suzuki.k.db@m.titech.ac.jp (K.S.); iwasaki.t.aj@m.titech.ac.jp (T.I.); 3 Department of Civil and Environmental Engineering, Tokyo Institute of Technology, Meguro, Tokyo 152-8552, Japan; hekizono.a.aa@m.titech.ac.jp (A.H.); iwanami@cv.titech.ac.jp (M.I.)

**Keywords:** negatively charged nitrogen-vacancy (NV) center in diamond, magnetometry, quantum sensing, gradiometer configuration, variable base length

## Abstract

The negatively charged nitrogen-vacancy (NV) center in diamonds is known as the spin defect and using its electron spin, magnetometry can be realized even at room temperature with extremely high sensitivity as well as a high dynamic range. However, a magnetically shielded enclosure is usually required to sense weak magnetic fields because environmental magnetic field noises can disturb high sensitivity measurements. Here, we fabricated a gradiometer with variable sensor length that works at room temperature using a pair of diamond samples containing negatively charged NV centers. Each diamond is attached to an optical fiber to enable free sensor placement. Without any magnetically shielding, our gradiometer realizes a magnetic noise spectrum comparable to that of a three-layer magnetically shielded enclosure, reducing the noises at the low-frequency range below 1 Hz as well as at the frequency of 50 Hz (power line frequency) and its harmonics. These results indicate the potential of highly sensitive magnetic sensing by the gradiometer using the NV center for applications in noisy environments such as outdoor and in vehicles.

## 1. Introduction

The negatively charged nitrogen-vacancy (NV) center in diamond is regarded as a promising quantum sensor candidate by which sensing with extremely high sensitivity and high spatial resolution can be realized because of its excellent optical and spin properties, such as bright single-photon emission, spin-dependent luminescence intensity and long coherence time even at room temperature [1,2,3]. Since the sensitivity increases with increasing amounts of the NV center, an extremely weak magnetic field can be measured using the NV center ensembles. It is also possible to resolve the nanometer level of the spatial resolution of the magnetic field using a single NV center. In addition, magnetic-field sensing can be carried out even in high magnetic field regions (more than ten Tesla) since signals do not saturate [4]. Thus, this indicates that we can realize magnetic field sensors with both high sensitivity and wide dynamic range using the NV center. Moreover, quantum-sensing based on the NV center can be applied to a wide variety of fields since diamond is stable against harsh environments [5,6,7] and is biocompatible [8,9,10]. In a previous study, Barry et al. detected magnetic fields from neuronal action potential of marine worms and squid using an NV layer created near the surface of the diamond [11]. For a system of applications, compact integrated magnetometers using the NV center demonstrated the minimal detectable magnetic field of sub−10 nT/√Hz sensitivity over a 125 Hz bandwidth using a balanced detection method [12] and 1 μT in 1 ms integration time with the specific construction volume of about 2.9 cm^3^ [13]. For the sensitivity improvements, the magnetic sensitivity of 0.9 pT/√Hz was achieved for an AC magnetic signal at 20 kHz [14] and magnetic signals in the frequency range between 10 Hz and 1 kHz [15]. As a result of these epoch-making demonstrations of magnetometry using the NV center, magnetoencephalography (MEG) [16], which requires highly sensitive magnetic sensors, is now one of the most challenging target applications using the NV center.

Currently, a superconducting quantum interference device (SQUID) [17,18] and an alkali vapor cell [19,20] are used for highly sensitive magnetic-field sensing to detect extremely small signals such as MEG signals. However, magnetic shield boxes/rooms are usually needed to realize the magnetic-field sensing because environmental magnetic noise disturbs the sensitive sensors needed to detect small signals [21,22]. A gradiometer is a method to reduce the effect of the magnetic noise when there are not any magnetic shield boxes/rooms [21,23,24,25]. Using the gradiometer configuration that uses multiple magnetometers to measure the gradient magnetic field, it is possible to remove environmental magnetic noises because the differential signal at two detection positions cancels a spatially homogeneous magnetic field. For gradiometers using the NV center, Shin et al. reported that the sensitivity of 4.6 ± 0.3 nT/√Hz was achieved using the gradiometer technique with a base length of 300 μm [26]. Here, the “base length” is defined as the distance between the two detection points. It was also reported that several micrometer scale spatial resolutions of magnetic field variations of the order of 10 μT were demonstrated using diamond connected to a dual-core photonic crystal fiber for the gradiometer configuration [27]. Ideally, to detect small signals efficiently, the optimum base length should be selected depending on the distance between the measurement target and sensors, the sensor sensitivity, and the inhomogeneity of magnetic noise. In general, to improve the signal-to-noise ratio (SNR), the base length should be longer than the distance between the measuring object and sensors. However, in previous studies, two fixed detection points in a diamond chip were used for the gradiometers. Thus, the distance between each point was the length of the order of micrometers. On the other hand, when we apply the gradiometer methodology to MEG, the base length should be of the order of a centimeter or more. It is also worth mentioning that we can expand the NV center magnetometry not only inside of rooms but also outdoors if the NV center gradiometer method is developed.

In this study, we fabricated the NV gradiometer with the variable base length using a configuration of optical fibers and a pair of diamonds separated from a diamond in which a high concentration of the NV centers was created by electron irradiation. The gradiometer demonstrated the high sensitivity of magnetic field measurement compatible with that in a magnetically shielded enclosure consisting of three-layer permalloy shield boxes. Furthermore, our gradiometer placed at 50 mm apart from the target detected a magnetic signal with a base length up to 100 mm, which is important for sensing a deep signal, such as the brain. Our results are a crucial step towards MEG working at room temperature without the requirement for magnetic shielding.

## 2. Materials and Methods

**Figure 1:**
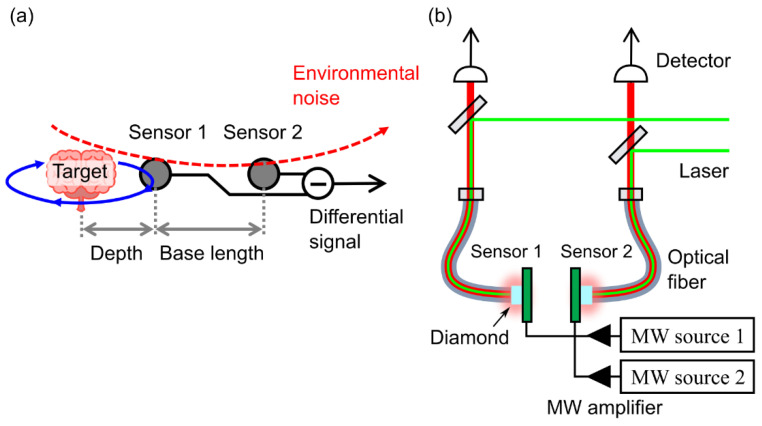
(**a**) Schematic representation of the gradiometer setup. A longer base length than the depth enabled us to selectively detect the target signal. (**b**) A schematic setup of the gradiometer using the nitrogen-vacancy (NV) centers in a pair of diamonds. A dichroic mirror selectively reflected the laser beam and transmitted the fluorescence from NV centers. The fluorescence from each diamond (sensor 1 and sensor 2) was detected by the respective photodiode connected to an oscilloscope.

A gradiometer cancels a homogeneous magnetic field by subtracting the signal at sensor 2 from the signal at sensor 1 (Figure 1a). A local magnetic signal that is the target signal decays quickly, whereas a noise coming from afar decays gradually. Thus, the gradiometer cancels the environmental magnetic noise that limits highly sensitive magnetic sensing. To realize sensing with high sensitivity, the base length is the important parameter for the gradiometer. Thus, the distance between the target and sensor 1, called “depth”, should be minimized for highly sensitive sensing, although the base length should be longer than the depth. For example, for sensing a deep brain signal, a base length of 50 mm or more is preferable.

Figure 1b shows a schematic setup of the gradiometer using two diamond quantum sensors that enabled us to change in a wide range of the base length. In each sensor, each diamond sample was attached to an optical fiber is mounted on a coplanar waveguide antenna that controls the NV centers homogeneously. The antenna had a similar geometry as in a previous study [28], but it was not a resonator type for operating spins in the NV centers using a wide frequency range. An amplifier was connected to the coplanar-waveguide antenna via a coaxial cable to amplify the continuous-wave from the microwave (MW) source. The microwave with 25 dBm was frequency modulated at 2 kHz with a frequency deviation width of 8 MHz. A 532 nm laser beam with 300 mW of optical power through the optical fiber illuminated both diamond samples containing the NV centers. Fluorescence from NV centers in a volume of 1.2 mm^3^ in each diamond was collected by a photodetector (Thorlabs FDS100A2) through the optical fiber, and photons detected by photodetectors were digitized using an oscilloscope (Keysight Technologies DSOS054A). A dichroic mirror and a long pass filter selectively passed through the fluorescence at wavelengths above 600 nm. The data sampling rate and collection time were 25 MS/s and 15 s, respectively. Then, a computer extracted the modulated frequency component from the fluorescence signals collected by the oscilloscope. Finally, the signals subtracting the signals at sensor 2 from the signals at sensor 1 were obtained. The magnetic field strength was obtained from the change in the optically detected magnetic resonance (ODMR) signal.

**Figure 2:**
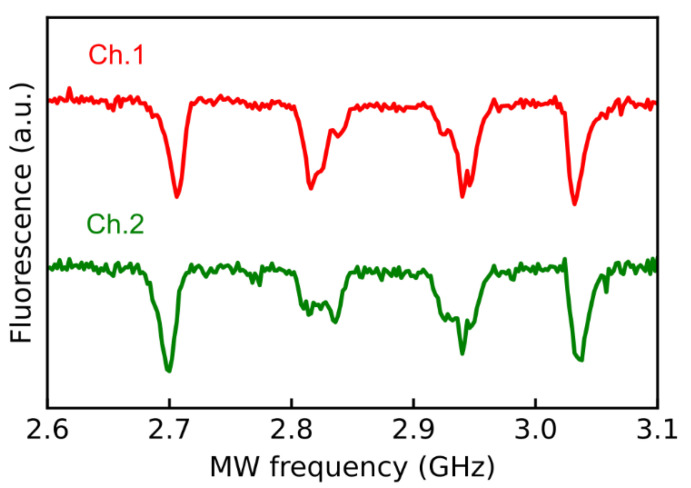
Typical optically detected magnetic resonance (ODMR) spectra of the spatially isolated diamond samples of sensor 1 (Ch. 1) and sensor 2 (Ch. 2). The detected fluorescence at each detector is about 0.2 mW. The ODMR signals (dips) of the left and right sides correspond to magnetic sublevels m_s_ = −1 and +1, respectively, for the NV center with [111] direction. The resonance of the other three of the four orientation axes is degenerate. Our microwave circuit drives the NV center with [111] direction most effectively. For each sensor, the noise voltage of the ODMR measurement is 0.057% of the signal voltage of the fluorescence.

Typical ODMR signals observed from sensors 1 and 2 are shown in Figure 2. A bias magnetic field of 2 mT was applied by a magnet during the measurement to separate the magnetic sublevel. The ODMR signals showed different intensities, as shown in Figure 2 since the NV centers in diamond have four orientation axes and can detect only the component of the magnetic field parallel to the orientation axis. We used the strongest ODMR signals to measure the magnetic field in this study, i.e., the most left side of the ODMR signals shown in Figure 2. The line widths of the ODMR resonance of Ch.1 and Ch.2 were 12.8 MHz and 13.1 MHz with a contrast of 1.7% and 1.8%, respectively. These diamonds had similar quantum properties because an electron irradiated diamond sample was divided into two parts, as explained below. Since we used two MW sources, the microwave frequency could be adjusted independently at the steepest point of each ODMR spectrum of the magnetic sublevel m_s_ = −1 in the following experiments. Our gradiometer obtained the difference between the magnetic field strength at each sensor. The magnetic field strength along the orientation axis was calculated from the fluorescent signal at the steepest point of each ODMR spectrum. Details are described in Appendix A.

**Figure 3:**
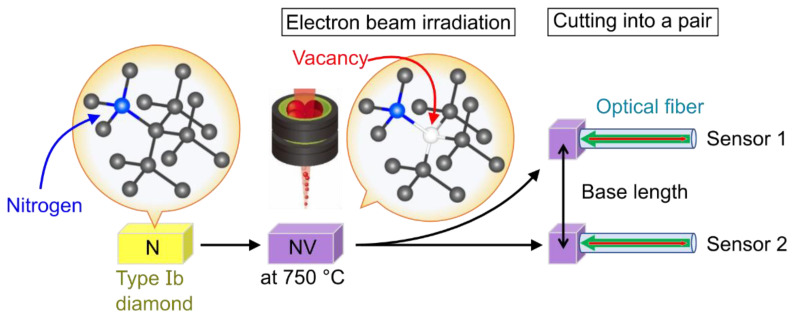
Fabrication process of the NV centers in diamond using electron beam irradiation. A type Ib diamond after electron beam irradiation is divided into two and attached to each optical fiber.

Next, the diamond sample used in this study is briefly mentioned. Our gradiometer used the spatially isolated equal-quality diamond pair to realize measurements with variable detection points. Figure 3 shows the fabrication process of the diamond pair from the type Ib diamond, which contains a nitrogen concentration of more than 10^19^ atoms/cm^3^. The Ib diamond was irradiated with 2 MeV-electrons at a fluence of 1 × 10^18^ /cm^2^. To avoid the accumulation of crystal damage and create the NV centers during irradiation, the electron irradiation was carried out at 750 °C [29]. The gradiometer configuration required diamonds with equal-quality properties such as quantum coherence time, the number of the NV centers. Therefore, the exposed diamond was split into two pieces.

## 3. Results and Discussion

### 3.1. Cancellation of a Spatially Homogeneous Magnetic Field Noise

**Figure 4:**
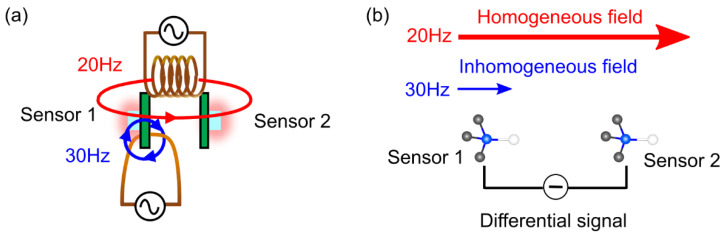
(**a**) Gradiometer with the solenoid coil and the copper wire. The base length of the gradiometer was 27 mm. (**b**) Direction of the magnetic fields and the orientation axis of the NV center.

To confirm the cancellation of the magnetic noise by the gradiometer, the following experiment was carried out. First, the spatially homogeneous AC magnetic field of 20 Hz was induced by a solenoid coil as the environmental magnetic field noises (Figure 4a). The magnetic field of 31 μT was generated by the solenoid coil with a current of 2A. Then, the in-homogeneous AC magnetic field of 30 Hz, which was used as the targeted signal, was induced by a copper wire beside sensor 1. The wire with a current of 1A generated a magnetic field of 10 μT. Both AC magnetic fields were applied along the orientation axis of the NV center (Figure 4b).

**Figure 5:**
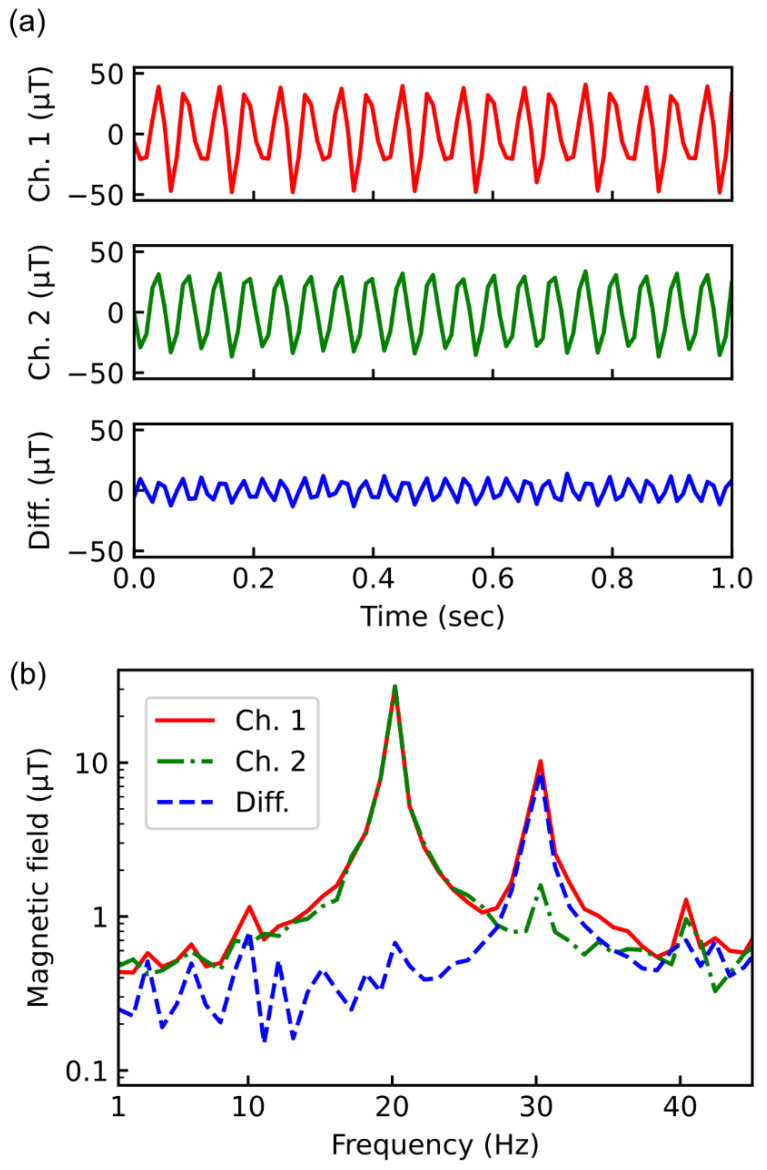
(**a**) Time-domain signals at sensor 1 (Ch.1), sensor 2 (Ch.2) and subtracted signal (Diff.). The detected wave for Ch. 2 is almost a single frequency wave because the copper wire applying a magnetic field of 30 Hz is far from sensor 2. On the other hand, since sensor 1 detects both 20 and 30 Hz magnetic fields, the detected wave at Ch.1 is a combination of the sin wave. (**b**) Fourier-transform of the time-domain signals from Ch.1 (solid red line), Ch. 2 (dashed-dotted green line) and diff (dashed blue line).

Figure 5 shows the time-domain signals of the detected magnetic field. The microwave sources for sensor 1 (Ch. 1) and sensor 2 (Ch. 2) adjust the frequency of the microwave to 2.71 and 2.7 GHz, respectively, which were the optimum points of the ODMR spectrum of the m_s_ = −1 spin state for sensor 1 and sensor 2. Sensor 1 detected the small spatially inhomogeneous magnetic field signal modulating the large spatially homogeneous magnetic field signal (Figure 5a, Ch. 1). Sensor 2 detected only the large spatially homogeneous magnetic field signal (Figure 5a, Ch. 2). The small spatially inhomogeneous magnetic field signal remained in the differential signal due to the cancellation of the large spatially homogeneous magnetic field signal (Figure 5a, diff.).

Fourier-transform of the time-domain signals indicated the ability the cancellation of the magnetic noise (Figure 5b). The spatially homogeneous AC magnetic field of 20 Hz was reduced to less than 1/50 in the differential signal. On the other hand, the in-homogeneous AC magnetic field of 30 Hz was detected by Ch. 1, whereas no significant peak was observed for Ch. 2. As a result, a peak at 30 Hz was observed in the differential signal. At 30 Hz, the subtracted signal (Diff.) was about 10% smaller than Ch.1 because Ch. 2 also detected small amounts of the component with 30 Hz. This indicated that the shorter the base length, the greater the loss of the difference signal. In the next section, the effect of base length on the loss is discussed.

### 3.2. Base Length Dependence

**Figure 6:**
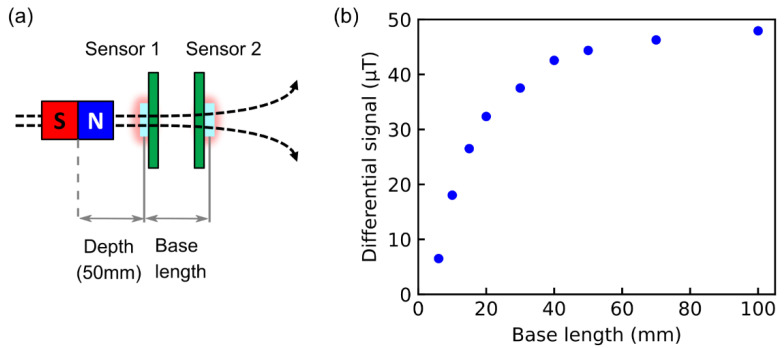
(**a**) Setup for measuring the base length dependence. Sensor 1 and sensor 2 are connected to optical fibers independently, allowing to change the base length freely. (**b**) Calculated differential signal level using the gradiometer as a function of the base length.

As above-mentioned, the distance of the base length to the target depth is a key parameter for magnetometry (noise cancelation) by gradiometer. We measured the base length dependence of the acquired signal. One sensor of the gradiometer was placed at 50 mm apart from the target magnet assuming the target object is in a deep part of the body, such as in the brain. By varying the position of the other sensor, i.e., the base length, we measured the differential signal level. The results of Figure 6 clearly demonstrated that the differential signal dramatically increased with increasing base length, and the saturation was observed at a base length above 50 mm. This result indicates that the gradiometer with variable base length demonstrated in this study is useful to sense a target with deep regions. To obtain large signals of the gradiometer, the base length should be longer than the depth. On the other hand, to cancel the magnetic noise, sensor 1 and sensor 2 should detect the same level. Therefore, the base length should be set appropriately, considering the distance between the measurement target and the noise source. In this respect, the gradiometer using the optical fiber has an advantage since the base length can be easily changed without the adjustment of the optical path.

### 3.3. Comparison between Gradiometor and Magnetically Shielded Enclosure

**Figure 7:**
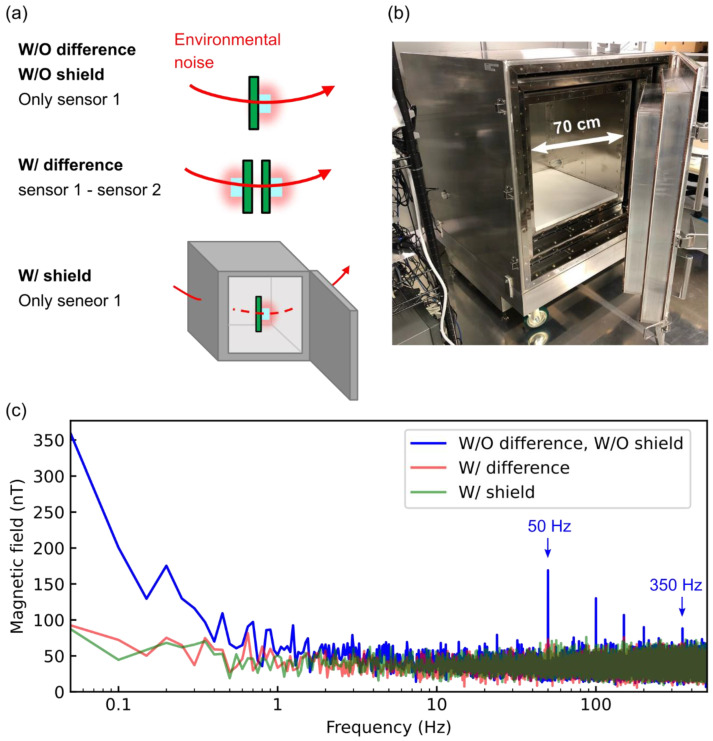
(**a**) Setup for measuring the magnetic noise frequency spectrum of the environment. (W/O difference, W/O shield) Only sensor 1 without any magnetic shielding nor using the gradiometer. (W/difference) The gradiometer with the base length of 50 mm. (W/ shield) Only sensor 1 in the three-layer magnetically shielded enclosure. (**b**) Three-layer magnetically shielded enclosure. The size of 70 cm is for magnetic sensing of small animals inside the enclosure. (**c**) Magnetic noise frequency spectrum. The peaks between 50 and 350 Hz are from the commercial power supply (50 Hz) and its harmonics. These magnetic noises propagated in space because the signal at 50 Hz and its harmonics disappear in a magnetically shielded enclosure (W/shield).

The effectiveness of the noise cancelation is shown in Figure 7. We compared the environmental magnetic noise between the gradiometer, a single sensor in a magnetically shielded enclosure, and the single sensor without any magnetic shielding (Figure 7a). The magnetically shielded enclosure consisted of three-layer permalloy shield boxes that had a shielding performance of more than 60 dB for fluctuating magnetic fields above 1 Hz (Figure 7b). In the case of no magnetic shielding nor gradiometer, the magnetic noise was found, which limited the highly sensitive magnetic sensing (Figure 7c). The frequency peaks corresponded to the frequency of the utility frequency, i.e., 50 Hz, and its harmonics. One of the magnetic noises at low frequency came from the trains running beside our building. In the case of using the gradiometer, the noise floor was 34 nT, which was comparable to that observed in the magnetically shielded enclosure. The gradiometer canceled magnetic noises at the frequency of the utility frequency and its harmonics. Moreover, the gradiometer reduced magnetic noised to the same level as that in the magnetically shielded enclosure. For the frequency below 1 Hz, the noise level of the magnetically shielded enclosure (W/shield) and gradiometer (W/difference) was remarkably reduced compared to the only sensor 1 without the magnetically shielded enclosure (W/O difference, W/O shield). Thus, the gradiometer successfully removed the magnetic noise at the same level as the magnetic enclosure. However, the noise floor above 10 Hz was the same for all three methods because of the limitation of the sensor sensitivity. For further sensitivity improvement, one of the limiting factors was the fluctuation of the resonance frequency of the NV center due to thermal drift. The double quantum method that used two spin states m_S_ = ±1 cancels this fluctuation [15,30,31]. We intend to apply the double quantum method as a further investigation. Moreover, the magnetically shielded enclosure was not good at removing low-frequency magnetic noise. Therefore, it was worth mentioning that the gradiometer had more capability to remove noises in low-frequency regions when the quality of the NV center was improved.

## 4. Conclusions

We demonstrated the gradiometer with variable base length using a pair of diamonds containing the NV center and evaluated the gradiometer configuration and performance. An applied spatially homogeneous magnetic field was reduced to less than 1/50 in the differential signal. As a result, a signal at 10 μT with a frequency of 30 Hz was detected in the magnetic noise at 31 μT with a frequency of 20 Hz by the gradiometer. Using the gradiometer, the background magnetic noise was reduced to the same level as that in the magnetically shielded enclosure in the frequency range below 1 Hz. In addition, the gradiometer removed the magnetic noise of the commercial power supply frequencies of 50 Hz and its harmonics. Thus, the result suggests that the NV center gradiometer can be applied to high-sensitive magnetometry even without any magnetically shielded enclosure, such as outdoor application for exploration of natural resources and monitoring the consciousness of car drivers. Since the gradiometer adopts the optical fiber system, the gradiometer is suitable for constructing a portable system, not only the flexibility of the base length depending on the measurement target and magnetic field environment. To realize gradiometer applications, further investigations to improve the magnetic sensitivity are necessary. We intend to create the NV center ensembles with high-quality, e.g., high concentration NV with long spin coherence time, high ODMR contrast, etc., by optimizing electron irradiation and thermal treatment conditions. In addition, to realize a field-portable diamond NV gradiometer with extremely high sensitivity, we will optimize the design of the gradiometer, including the improvement of the spin manipulation sequence.

## Figures and Tables

**Figure A1:**
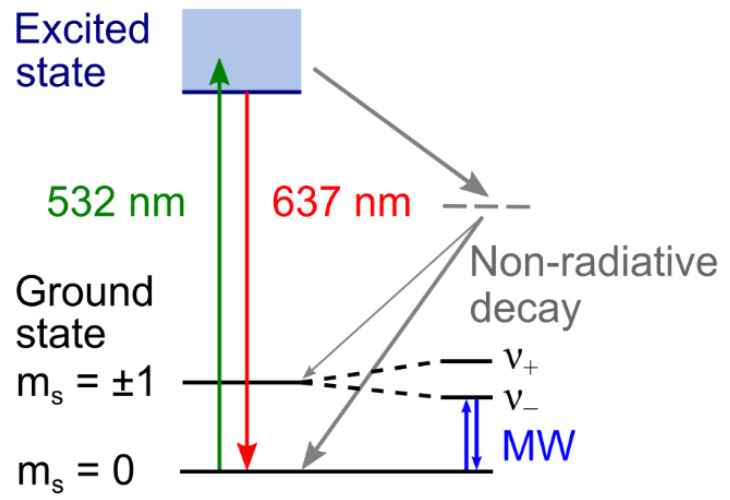
Energy-level diagram of the negatively charged NV center in diamond. The two magnetic sublevels with m_S_ = ±1 split due to the Zeeman effect. The zero phonon line of the NV center is at 637 nm.

## Data Availability

The data that support the findings of this study are available within this article and also from the corresponding author on request basis.

## Appendix A

Magnetic sensing by the NV center is achieved by measuring the resonance frequency of magnetic resonance. The shift of the resonance frequency of the NV center by the magnetic field B parallel to the orientation axis of the NV center is:

(A1) $$\nu_{\pm }\;=\;D\;\pm \;g\mu_{B}\;B/h,$$

where zero-field-splitting parameter D ≈ 2.87 GHz, g$\mu_{B}$/h = 28 GHz/T is the gyromagnetic ratio of the electronic spin (Figure A1). Since the intensity of fluorescence reflects the magnetic level of the NV center, the magnetic resonance spectrum is obtained optically. Measuring the quantum state by light achieves a highly sensitive measurement because the energy of light is sufficiently higher than that of the environment.

Compared to a SQUID pickup coil gradiometer, our gradiometer used a different method to cancel the homogeneous magnetic signal. In the case of the SQUID pickup coil, the constitution of the pickup coils was designed to obtain the subtracted signal by canceling the current from each pickup coil [32]. While the NV center had a high dynamic range, the subtracted signal was calculated by the computer after performing the magnetic measurement.
